# Integrated metabolite profiling and molecular docking reveal anti-aging potential of *Clerodendrum infortunatum* L. fractions

**DOI:** 10.1038/s41598-026-47614-3

**Published:** 2026-04-22

**Authors:** Fatma Atef, Mostafa A. Abdelkawy, Basma M. Eltanany, Laura Pont, Ahmed A. Al-Karmalawy, Fernando Benavente, Inas Y. Younis, Asmaa M. Otify

**Affiliations:** 1 Postgraduate Program in Department of Pharmacognosy, Faculty of Pharmacy, Cairo University, Cairo, 11562 Egypt; 2 Boulaq El-Dakrour General Hospital, Giza, 12617 Egypt; 3 Department of Pharmacognosy, Faculty of Pharmacy, Cairo University, Cairo, 11562 Egypt; 4 Department of Pharmaceutical Analytical Chemistry, Faculty of Pharmacy, Cairo University, Cairo, 11562, Egypt; 5 Department of Chemical Engineering and Analytical Chemistry, Institute for Research on Nutrition and Food Safety (INSA·UB), University of Barcelona, Barcelona, 08028, Spain; 6 Serra Húnter Program, Generalitat de Catalunya, Barcelona, 08007, Spain; 7 Department of Pharmaceutical Chemistry, College of Pharmacy, The University of Mashreq, Baghdad, 10023, Iraq; 8 Department of Pharmaceutical Chemistry , Faculty of Pharmacy, Horus University, New Damietta, 34518, Egypt

**Keywords:** Anti-collagenase, Anti-elastase, Chemometrics, *Clerodendrum infortunatum*, LC-MS/MS, Metabolomics, Molecular docking, Biochemistry, Computational biology and bioinformatics, Drug discovery, Plant sciences

## Abstract

Skin aging is a complex and irreversible natural process impacted by genetics, lifestyle, and environmental variables, leading to wrinkles, loss of elasticity, and uneven skin tone. There is a growing demand for natural skincare products, which are perceived as safer, more sustainable, and effective in combating the signs of aging. *Clerodendrum infortunatum* (*C. infortunatum*) Linn. (Family Lamiaceae) has traditional medicinal uses, including hepatoprotection, antimicrobial, and vermifuge activity, as well as benefits in alleviating inflammation, arthritis, and diabetes. This study aims to investigate the metabolites present in different solvent-extracted fractions of the aerial parts of *C. infortunatum* through liquid chromatography-tandem mass spectrometry (LC-MS/MS) profiling, and to evaluate their anti-aging potential using *in vitro* anti-collagenase and anti-elastase assays. The correlation between the identified metabolites and bioactivity was investigated using a partial least squares (PLS) chemometric approach. To confirm these findings, molecular docking studies were performed to assess the inhibitory potential of the metabolites most strongly correlated with bioactivity against elastase and collagenase target enzymes. This study is the first to correlate the secondary metabolites of *C. infortunatum* solvent-extracted fractions with their newly discovered anti-aging properties, representing a significant contribution to the field. Additionally, it presents the first molecular docking analysis of salsaside A, jionoside C, and 6′-caffeoyl-12-glucosyloxy-jasmonic acid against collagenase and elastase enzymes, revealing significant binding affinities and promising inhibitory potential. These findings underscore the potential of these metabolites as novel anti-aging compounds through their strong interactions with key target enzymes.

**Supplementary Information:**

The online version contains supplementary material available at 10.1038/s41598-026-47614-3.

## Introduction

Aging is an inescapable natural phenomenon where the body undergoes gradual degradation of its components, tissues, and functions. The skin aging process has been divided into two categories, intrinsic and extrinsic aging. Natural or intrinsic aging is caused by changes in the elasticity of the skin over time^1^. It is primarily driven by genetic factors and internal physiological changes. Extrinsic skin aging results from exposure to solar radiation (photoaging), chemicals, nicotine, and pollutants, which lead to the generation of reactive oxygen species (ROS)^2^.

The extracellular matrix in the skin is a complex network of proteins, primarily composed of collagen, elastin, proteoglycans, and glycoproteins, which provides structural support and maintain skin strength, elasticity, and hydration^3^. Elastin allows the skin to stretch and return to its original shape, while collagen, the most abundant protein in the dermis, offers tensile strength. Both are crucial for preserving skin structure and youthful appearance. Importantly, intrinsic and extrinsic factors can increase the expression of elastases and matrix metalloproteinases (MMPs), including collagenases, key enzymes responsible for degrading connective tissue and elastic fibers. MMPs, which are zinc-dependent endopeptidases, are essential for extracellular matrix degradation, with collagenases (MMP-1, MMP-8, MMP-13, and MMP-18) specifically targeting collagen during the photoaging process. On the other hand, elastase degrades elastin, leading to a loss of skin elasticity. The increased activity of these enzymes leads to visible signs of aging, such as wrinkles and sagging skin^4,5^.

Synthetic ingredients are often deemed undesirable due to their potential to cause allergic reactions. In contrast, natural products, which have been utilized for generations in skincare, are becoming increasingly popular among consumers. This growing preference has increased demand for the discovery and development of novel natural products for use in skincare and cosmetic products^5^.

The Lamiaceae family is recognized as one of the most significant medicinal plant families utilized in skincare products, due to its antioxidant and antibacterial properties^6^. *Clerodendrum infortunatum* (*C. infortunatum*, syn. *C. viscosum*) is a member of the Lamiaceae family. This shrub is native to India and North Bengal and is widely distributed in subtropical and tropical regions worldwide. Its rich content of bioactive compounds, including secondary metabolites such as triterpenes, steroids, flavonoids, and polyphenols, has stimulated research demonstrating its antioxidant, anti-inflammatory, analgesic, antibacterial, antimalarial, and anthelmintic properties^7,8^. To support these *in vitro* and in vivo bioactivity investigations, untargeted metabolomics based on liquid chromatography-quadrupole time-of-flight tandem mass spectrometry (LC-QTOF-MS/MS) has proven effective for comprehensive metabolite profiling of medicinal and culinary plants^7,9–11^. While the Lamiaceae family is traditionally recognized for its essential oils and volatile constituents, this study focuses on non-volatile metabolites analyzed *via* LC–MS/MS, aiming to uncover additional bioactive compounds that may contribute to skincare benefits.

This study presents metabolite profiling of fractions derived from a total methanolic extract of the aerial parts of *C. infortunatum*, analyzed in negative ionization mode. The fractions were obtained using solvents of varying polarities, namely, hexane (HEX), dichloromethane (DM), ethyl acetate (EA), and *n*-butanol (BU), to investigate the metabolites potentially responsible for anti-aging activity. This activity was investigated *in vitro *through collagenase and elastase inhibition assays. Chemometrics provided a valuable tool for multivariate data analysis of metabolite profiles, enabling the identification of fraction metabolites most strongly correlated with anti-aging properties. To our knowledge, this is the first study to link the secondary metabolites of *C. infortunatum* fractions with their newly discovered anti-aging effects. Additionally, the metabolites most strongly correlated with this bioactivity were further evaluated using molecular docking, a computational method that predicts the binding affinity of bioactive compounds to target proteins^9,12,13^. This approach revealed compounds with potentially stronger affinities for collagenase and elastase enzymes than standard reference drugs.

## Materials and methods

### Plant material and preparation of plant extracts

*C. infortunatum* aerial parts were collected from Mazhar Botanical Garden in El Bragil, Egypt. The plant samples were identified by Agriculture Engineer Therese Labib, a plant taxonomy consultant with the Ministry of Agriculture in Giza, Egypt. The plant materials were collected with permission and in accordance with national requirements from the Agricultural Research Center, Giza, Egypt (9 Cairo University Road, Giza District, Giza Governorate). A voucher specimen was deposited at the Herbarium of the Pharmacognosy Department, Faculty of Pharmacy, Cairo University, Cairo, Egypt (voucher No.10.3.2021). After air-drying, the aerial parts were ground into powder using a powder grinder. At room temperature, five kg of dried powder was exhaustively extracted using 85% v/v methanol/water (25 L). Hydroalcoholic mixtures (70–90% methanol) are widely reported to provide superior recovery of phenolics, flavonoids, alkaloids, glycosides, and other moderately polar secondary metabolites due to enhanced plant matrix swelling and improved mass transfer^14^. The extract was filtered through a filter paper (Whatman No. 1) and concentrated to dryness under vacuum at 45 °C using a rotary evaporator (BUCHI Rotavapor R-300, Cole-Parmer, Vernon Hills, USA), resulting in 500 g of total extract. Subsequently, 400 g of the dry extract was suspended in 350 mL of distilled water and successively partitioned with solvents of increasing polarity, HEX, DM, EA, and BU (each 500 mL × 6), until depletion, as indicated by a colorless organic phase. Sequential partitioning with solvents of increasing polarity was used to fractionate metabolites, allowing enrichment of non-polar (HEX), low- to medium-polar (DM), semi-polar (EA), and polar (BU) compounds for further analysis^15^. The fractions were pooled separately, and the organic solvents were evaporated using the rotary evaporator, yielding 100 g, 3 g, 12 g, and 30 g for HEX, DM, EA, and BU, respectively. All chemicals were purchased from Piochem Company for Pharmaceuticals and Chemicals (Cairo, Egypt). Biological triplicates were prepared and extracted in parallel under identical conditions.

### LC-QTOF-MS/MS metabolite profiling

The samples were prepared for LC-QTOF-MS/MS analysis by sonicating 10 mg of each dried fraction in 1 mL of methanol, centrifuging at 13,000×*g* for 10 min, and filtering through a 0.22 μm syringe nylon filter. The analyses were conducted using a 1260 Infinity LC system (Agilent Technologies, Waldbronn, Germany) coupled with an orthogonal electrospray ionization (ESI) interface to a 6546 LC/QTOF/MS/MS. Metabolite profiling was performed in negative ESI mode by injecting 5 µL of sample into a Zorbax SB-C18 column (5 μm, 150 mm × 2.1 mm), using an optimized acetonitrile–water gradient system, with both solvents containing 0.1% (v/v) formic acid. The chromatographic, MS, and MS/MS conditions, as well as data processing procedures, were as described in our previous study^16,17^. All solvents used were LC-MS grade and supplied by Merck (Darmstadt, Germany).

The MassHunter program was used for instrument control, data collection, and processing (Agilent Technologies). Retention time (R_t_), accurate molecular mass, predicted molecular formula, and MS/MS spectra were used for the identification of secondary metabolites. Identification was also confirmed by comparing with published literature and accessible databases, e.g., the Human Metabolome Database (http://www.hmdb.ca/), PubChem (https://pubchem.ncbi.nlm.nih.gov/), and the Phytochemical Dictionary of Natural Product Database (https://dnp.chemnetbase.com/faces/chemical/ChemicalSearch.xhtml). Consequently, the annotated metabolites were tentatively identified with a high level of confidence (probable structure, level 2: MS, MS/MS, and bibliography/database)^18^.

### Anti-aging activity

#### Anti-collagenase activity

The spectrophotometric assay was conducted in accordance with the manufacturer’s instructions, using the ab196999 Collagenase Activity Assay Kit (Abcam, Cambridge, UK). This method evaluates collagenase activity by utilizing a synthetic peptide substrate (FALGPA), which simulates collagen structure. In summary, aliquots of the test fractions and piroxicam (Pfizer, Cairo, Egypt), serving as the reference drug, were prepared in phosphate-buffered saline at varying concentrations. For each reaction, 2 µL of sample was combined with 10 µL of collagenase and 88 µL of assay buffer. The reaction was initiated by adding 40 µL of FALGPA and 60 µL of buffer. Absorbance was measured at 450 nm over a time interval of 5–15 min. The blank was prepared by replacing the test samples with solvent. All experiments were performed in triplicate. Results were expressed as mean IC_50_ (the concentration causing 50% inhibition of enzyme activity) ± standard deviation (SD):

$$\% {\mathrm{Inhibition}}\, = \,{\mathrm{1}}{-}\left[ {{\mathrm{Enzyme}}\,{\mathrm{activity}}\,{\mathrm{of}}\,{\mathrm{test}}\,{\mathrm{sample}} \div {\mathrm{Enzyme}}\,{\mathrm{activity}}\,{\mathrm{of}}\,{\mathrm{blank}}} \right] \times {\mathrm{1}}00$$

#### Anti-elastase activity

Porcine pancreatic elastase was employed to evaluate elastase inhibition, using Dye-Quenched elastin as the substrate for fluorescence-based detection. The assay was conducted according to the manufacturer’s instructions using the E-12,056 EnzChek Elastase Assay Kit (Abcam). Briefly, in a 96-well plate, 100 µL of diluted porcine pancreatic elastase was incubated with 50 µL of various test fractions, along with daidzein (Pfizer, Cairo, Egypt) as the reference drug, at different concentrations in Tris-HCl buffer (pH 8), in the dark at 25 °C for 30 min. After preincubation, 50 µL of Dye-Quenched elastin substrate solution was added to each well, resulting in a final reaction volume of 200 µL. The microplate was further incubated in the dark at 25 °C for 120 min. Background fluorescence was corrected by subtracting the fluorescence values of the no-enzyme control at each time point. All experiments were conducted in triplicate, measuring fluorescence with a microplate reader at 450 nm. Results were expressed as mean IC_50_ ± SD.

#### Statistical analysis

GraphPad Prism 5 (GraphPad Software, San Diego, USA) was used for statistical analysis of the anti-collagenase and anti-elastase assay data. One-way ANOVA and post-hoc multiple t-tests with Tukey’s correction were applied to assess statistical significance.

### Multivariate data analysis

#### Data preprocessing

MassHunter raw LC-MS chromatograms were converted into mzXML format using MSConvert 3.0 software (www.proteowizard.org) and processed with MZmine 2.53 (www.mzio.io/). A baseline cut-off method was used to select ion peaks, which were then subjected to a chromatogram builder and chromatogram deconvolution. The gap-filling peak finder was used to identify any missing peaks following deisotoping of the selected peaks using the isotopic peak grouper. The settings used for both the isotopic peak grouper and gap-filling were as follows: *m/z* tolerance of 0.05 *m/z* or 10 ppm, R_t_ tolerance of 0.2 absolute (min), and a minimum standard intensity of 5 × 10^3^.

Peak lists were aligned using the join aligner, and the resulting aligned peak list was exported as a CSV file (Excel 2016, Microsoft®, Redmond, WA, USA), generating a data matrix with information from all samples (four fractions analyzed in triplicate). The matrix included scan number, R_t_, *m/z*, and peak area for each detected compound, excluding features with *m/z* values below 100. From this matrix, 67 metabolites were identified based on accurate mass, R_t_, and MS/MS fragmentation data. Their peak areas were compiled into a refined matrix with 12 columns (triplicates of the four fractions) and 67 rows (identified metabolites). This dataset was mean-centered and exported to SIMCA-P (version 14, Umetrics, Umeå, Sweden), where it was Pareto scaled prior to partial least squares (PLS) analysis^19^.

#### Partial least squares

PLS analysis was used to assess the correlation between the studied bioactivities and the abundances (i.e., peak area) of the identified metabolites (Suppl. Table S1). Metabolites significantly contributing to the observed bioactivities were identified based on their variable importance to projection (VIP) values obtained from the PLS model. Model validation was conducted using permutation tests and regression analysis. A correlogram plot was generated with RStudio (version 2022.07.1, Boston, USA), and correlation levels were determined using the following thresholds: negligible (*r* < 0.3), weak (*r* = 0.3–0.5), moderate (*r* = 0.5–0.7), strong (*r* = 0.7–0.9), and very strong (*r* = 0.9–1.0)^11,16^.

### Molecular docking

The open-source AutoDock Vina and PyMol softwares^20^ were used to investigate the inhibitory potential of metabolites strongly correlated with bioactivity against elastase and collagenase, using their respective enzyme structures (protein data bank (PDB) IDs: 4YM9 and 7Z5U, www.rcsb.org). The chemical structures of the metabolites were constructed using ChemDraw (Revvity, Inc, Waltham, MA, USA) and then subjected to partial charge optimization, 3D hydrogenation, and energy minimization^21^. The target enzyme structures were retrieved from the PDB and prepared through structure correction, 3D hydrogenation, and energy minimization^22^. Molecular docking was conducted for each enzyme-ligand pair, and the results were compared to those of the co-crystallized inhibitors. The docking protocol was validated by redocking the co-crystallized inhibitor of each target and obtaining low root-mean-square deviation RMSD values (< 2 Å)^13^.

## Results and discussion

### LC-QTOF-MS/MS metabolite profiling of *C. infortunatum* fractions

**Fig. 1:**
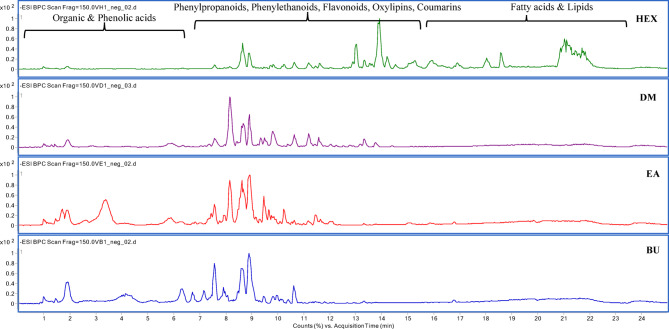
Representative LC-QTOF-MS/MS base peak chromatograms of the hexane (HEX), dichloromethane (DM), ethyl acetate (EA), and butanol (BU) fractions from the aerial parts extract of *C. infortunatum* in negative ESI mode.

The chemical profiles of the fractions obtained from the total extract of the aerial parts of *C. infortunatum* were analyzed using LC-QTOF-MS/MS in the negative ESI mode. Negative ESI mode is particularly suitable for the major metabolite classes targeted in this study, including organic acids, phenolic compounds, fatty acids, and other acidic or polar metabolites, which ionize more efficiently and reproducibly in negative mode. Additionally, negative ESI provides improved sensitivity and reduced background noise^23,24^. The components of the fractions were eluted within 24 min based on their polarity. A total of 67 metabolites were tentatively identified, as detailed in Table 1. Their abundances (i.e., peak area) across the different fractions are provided in Suppl. Table S1. These metabolites represented various classes, including 4 organic acids, 8 phenolic acids, 17 phenylpropanoids and phenylethanoids, 9 flavonoids, 1 coumarin, 3 oxylipins, 6 other aromatic compounds, 17 fatty acids and lipids, and 2 miscellaneous compounds. Representative base peak chromatograms of the four fractions are shown in Fig. 1, and the MS/MS spectra of some of the most relevant metabolites are displayed in Suppl. Fig. S1−S24. The interpretation of the mass spectra for selected representative metabolites from each class is discussed below.

**Table 1:** Identified metabolites in the HEX, DM, EA, and BU fractions from the aerial parts extract of *C. infortunatum*, analyzed by LC-QTOF-MS/MS in negative ESI mode.

Peak No.	*R*_t_ (min)	*m/z*	Molecular formula	Error (ppm)	MS/MS fragments (*m/z*)	Metabolite name	References
*Organic acids*							
2	1.16	133.0139	C_4_H_5_O_5_^–^	2.6	89.0235, **71.0134**	Malic acid	^16^
4	1.47	117.0189	C_4_H_5_O_4_^–^	3.7	**73.0291**	Succinic acid	^25^
7	1.91	191.0565	C_7_H_11_O_6_^–^	-2.0	111.0444, 129.0553, **101.0603**	Quinic acid	^25^
8	2.13	279.1076	C_11_H_19_O_8_^–^	3.4	101.0618, 89.0220, **59.0132**	2-Hydroxy-2-methyl butyric acid hexoside	^26^
*Phenolic acids*							
5	1.70	169.0138	C_7_H_5_O_5_^–^	2.6	**125.0239**, 107.0137, 79.0183	Gallic acid	HMDB0005807
9	2.58	167.0345	C_8_H_7_O_4_^–^	5.9	137.0252, **123.0446**, 109.0293, 105.0337	Vanillic acid	^25^
10	3.40	153.0188	C_7_H_5_O_4_^–^	3.8	**109.0292**, 91.0190	Protocatechuic acid	HMDB0001856
13	5.87	137.0240	C_7_H_5_O_3_^–^	3.0	**137**, 108.0210, 93.0338, 92.0264, 81.0340	Hydroxy-benzoic acid	^16^
18	7.28	457.1331	C_20_H_25_O_12_^–^	4.5	163.0385, **119.0494**, 61.1016	Coumaric acid pentosyl-hexoside	^26^
21	7.46	179.0351	C_9_H_7_O_4_^–^	-0.6	**135.0444**, 117.0496, 89.0391	Caffeic acid	^8^
28	8.43	167.0343	C_8_H_7_O_4_–	4.1	152.0110, 135.0463, **108.0211**	Dihydroxy-methyl benzoate	^16^
43	9.92	193.0501	C_10_H_9_O_4_^–^	2.7	161.0231, 134.0373, **133.0289**	Ferulic acid	^16^
*Phenylpropanoids and phenylethanoids*							
11	3.86	461.1654	C_20_H_29_O_12_^–^	2.3	315.1083, 135.0451, **113.0241**, 85.0292	Decaffeoyl-acteoside (Phenylethanoid glycoside)	^25^
15	6.40	487.1446	C_21_H_27_O_13_^–^	2.3	**179.0344**, 135.0448, 113.0235	Cistanoside F (Caffeoyl glycoside)	^25^
16	6.81	341.0865	C_15_H_17_O_9_^–^	3.8	179.0337, 161.0237, **135.0447**	Caffeoyl glucose	^27^
24	7.87	785.2486	C_35_H_45_O_20_^–^	3.0	623.2188, 477.1609, **161.0239**	Echinacoside (Caffeoyl phenylethanoid glycoside)	^28^
25	7.90	639.1917	C_29_H_35_O_16_^–^	2.1	621.1804, **179.0341**, 161.0234, 135.0443	β-Hydroxy-verbascoside (Caffeoyl phenylethanoid glycoside)	^25^
29	8.50	653.2071	C_30_H_37_O_16_^–^	2.5	179.0345, **161.0237**, 135.0445	Campneoside I (Caffeoyl phenylethanoid glycoside)	^25^
30	8.64	623.1976	C_29_H_35_O_15_^–^	0.87	461.1651, 315.1072,179.0340, **161.0238**, 135.0446, 133.0289	Verbascoside (Caffeoyl phenylethanoid glycoside)	^25^
31	8.82	755.2377	C_34_H_43_O_19_^–^	3.6	593.2061, 461.1694, **161.0228**	Forsythoside B (Caffeoyl phenylethanoid glycoside)	^25^
32	8.91	623.1978	C_29_H_35_ O_15_	0.55	461.1645, 315.1090, **161.0237**, 135.0447	Iso-verbascoside (Caffeoyl phenylethanoid glycoside)	^26^
33	9.05	607.2031	C_29_H_35_O_14_^–^	0.21	461.2890, **161.0238**, 145.0284, 133.0287	Lipedoside A-Ⅰ (Coumaroyl phenylethanoid glycoside)	^29^
34	9.17	637.2126	C_30_H_37_O_15_^–^	1.9	315.1048, 193.0504, **175.0393**, 160.0159	Leucosceptoside A (Feruloyl phenylethanoid glycoside)	^30^
35	9.17	785.2278	C_38_H_41_O_18_^–^	2.6	**623.1909**, 477.1399, 461.1647, 315.1049, 297.0767, 179.0345, 161.0230	Caffeoyl-verbascoside (Caffeoyl phenylethanoid glycoside)	^7^
36	9.30	503.1187	C_24_H_23_O_12_^–^	1.6	179.0342, **161.0236**, 135.0443	Dicaffeoyl hexoside	
41	9.76	577.1903	C_28_H_33_O_13_^–^	4.1	269.1019, 179.0352, **161.0240**, 133.0289	Salsaside A (Caffeoyl phenylmethanoid glycoside)	^31^
42	9.85	651.2286	C_31_H_39_O_15_^–^	2.2	193.0499, **175.0392**, 160.0159, 113.0242	Martynoside (Feruloyl phenylethanoid glycoside)	^8,32^
46	10.24	591.2071	C_29_H_35_O_13_^–^	2.1	179.0342, **161.0237**, 135.0446, 133.0289	Jionoside C (Caffeoyl phenylethanoid glycoside)	^30^
47	10.54	693.2397	C_33_H_41_O_16_^–^	0.44	193.0499, **175.0396**, 160.0162, 134.0367, 113.0243	Acetyl martynoside (Feruloyl phenylethanoid glycoside)	^30^
*Flavonoids*							
27	8.31	637.1759	C_29_H_33_O_16_	2.4	475.1436, 329.0864, 311.0759, **161.0237**, 109.0292	Rhamnazin hexoside rhamnoside	^26^
37	9.29	431.0971	C_21_H_19_O_10_^–^	2.9	**268.0364**, 151.0040	Apigenin hexoside	HMDB0041591
44	10.00	593.1310	C_30_H_25_O_13_^–^	-1.57	447.0935, **285.0397**, 119.0484, 59.0132	Kaempferol coumaroyl-hexoside	^10^
45	10.20	593.1320	C_30_H_25_O_13_^–^	3.3	**269.0448**, 179.0337, 161.0237, 135.0446, 133.0293	Apigenin caffeoyl-hexoside	PubChem CID 44,257,827
48	10.63	459.0925	C_22_H_19_O_11_^–^	1.7	283.0613, **268.0368**	Acacetin 7-glucuronide	^30^
49	10.78	577.1373	C_30_H_25_O_12_^–^	-3.7	**269.0443**, 145.0295,117.0339, 93.0331	Apigenin coumaroyl-hexoside	PubChem CID 44,257,855
50	10.89	299.0544	C_16_H_11_O_6_^–^	5.7	**284.0301**, 273.6576, 227.0351	4´-Methyl scutellrein	^26^
52	11.45	269.0458	C_15_H_9_O_5_^–^	-0.9	151.0031, 225.0557, **117.0342**	Apigenin	^8,26^
54	13.42	283.0605	C_16_H_11_O_5_^–^	2.5	**268.0353**, 211.0407	Acacetin	^8^
*Coumarins*							
20	7.35	177.0193	C_9_H_5_O_4_^–^	0.2	133.0288, **105.0342**	Esculetin	HMDB0030819
*Oxylipins*							
22	7.52	387.1650	C_18_H_27_O_9_^–^	2.7	225.1095, 207.1019, **59.0132**	12-Hydroxy-jasmonic acid glucoside	HMDB0040706
26	8.15	225.1127	C_12_H_17_O_4_^–^	2.4	207.1019, 202.2730, **59.0133**	12-Hydroxy-jasmonic acid	PubChem CID 5,497,122
38	9.46	549.1968	C_27_H_33_O_12_^–^	1.7	387.1655, 207.1022, 179.0340, **161.0239**, 135.0443, 133.0290, 59.0134	6’-Caffeoyl-12-glucosyloxy-jasmonic acid	^30^
*Other aromatic compounds*							
6	1.72	125.0234	C_6_H_5_O_3_^–^	8.1	**125.024**, 81.0341, 79.0187	Pyrogallol or phloroglucinol	HMDB0013674 HMDB0013675
12	5.18	285.0607	C_12_H_13_O_8_^–^	3.1	152.0116, 109.0288, **108.0211**	Catechol hexuronide	HMDB0240490
14	6.24	109.0295	C_6_H_5_O_2_^–^	0.0	**108.0212**, 81.0353, 53.0394	Catechol	^25^
17	7.04	151.0400	C_8_H_7_O_3_^–^	0.5	133.0662, 124.6446, **108.0214**	Methoxy-benzoic acid	^16^
19	7.42	135.0446	C_8_H_7_O_2_^–^	4.1	**135.0446**, 117.0347, 107.0501	Phenyl-acetic acid	^16^
23	7.58	121.0292	C_7_H_5_O_2_^–^	2.5	**92.0257**, 82.5389	Benzoic acid	^16^
*Fatty acids and lipids*							
39	9.56	187.0970	C_9_H_15_O_4_^–^	3.1	**125.0970**, 97.0654	Nonanedioic acid (Azelaic acid)	^16^
40	9.73	243.1240	C_12_H_19_O_5_^–^	-0.8	**225.1109**, 207.1019 163.1105, 136.0535	Trihydroxy-dodecadienoic acid	^16^
51	11.20	327.2168	C_18_H_31_O_5_^–^	2.7	291.1949, 229.1439, **211.1332**	Trihydroxy-octadecadienoic acid	^16^
53	11.62	329.2350	C_18_H_33_O_5_^–^	-5	311.2233, 229.1463, **211.1334**	Trihydroxy-octadecenoic acid	HMDB0038555
55	13.81	675.3588	C_33_H_55_O_14_^–^	1.4	**277.2156**, 89.0234, 59.0134	Dihexosyl monoacyl glycerol (18:3)	HMDB0041093
56	13.91	555.2832	C_25_H_47_O_11_S^–^	2.3	255.2319, 225.0068, 164.9857, **80.9649**	Sulfoquinovosyl monoacyl glycerol (16:0)	^16^
57	15.93	295.2277	C_18_H_31_O_3_^–^	0.57	**277.2163**, 229.9924	Hydroxy-octadecadienoic acid	^26^
58	17.45	409.2358	C_19_H_38_O_7_P^–^	0.6	255.2297, 152.9956, **78.9588**	Monoacyl phosphoglyceride (16:0)	^16^
59	17.87	435.2502	C_21_H_40_O_7_P^–^	3.5	281.2471, **152.9952**, 78.9587	Monoacyl phosphoglyceride (18:1)	^16^
60	18.58	277.2166	C_18_H_29_O_2_^–^	2.5	233.2241, 141.0904, **71.0133**	Octadecatrienoic acid (linolenic acid)	HMDB0001388
61	19.63	279.2322	C_18_H_31_O_2_^–^	2.7	**279.2323**, 96.9627	Octadecadienoic acid (linoleic acid)	^8^
62	19.69	843.5297	C_45_H_79_O_12_S^–^	0.1	843.5366, 281.2480, 225.0068, **80.9644**	Sulfoquinovosyl diacyl glycerol (18:2/18:1)	^16^
63	20.85	255.2323	C_16_H_31_O_2_^–^	3.0	169.5369, **90.8202**, 77.3264, 55.1581	Hexadecanoic acid	^33^
64	21.02	815.4975	C_43_H_75_O_12_S^–^	2.8	255.2329, 225.0070, **80.9648**	Sulfoquinovosyl diacyl glycerol (16:0/18:3)	^16^
65	21.04	281.2478	C_18_H_33_O_2_^–^	2.9	**250.9255**, 176, 96.9589	Octadecenoic acid (Oleic acid)	^8^
66	21.12	841.5126	C_45_H_77_O_12_S^–^	1.8	281.2489, 225.0065, 164.9854, **80.9648**	Sulfoquinovosyl diacyl glycerol (18:1/18:3)	^16^
67	21.90	819.5287	C_43_H_79_O_12_S^–^	1.3	255.2309, 225.0075, 164.9864, **80.9647**	Sulfoquinovosyl diacyl glycerol (16:0/18:1)	^16^
*Miscellaneous*							
1	1.00	341.1080	C_12_H_21_O_11_^–^	2.7	89.0238, 71.0135, **59.0131**	Disaccharide	^16^
3	1.32	128.0349	C_5_H_6_NO_3_^–^	3.2	93.4115, **69.1404**	Pyroglutamic acid	^16^

Bold *m/z* numbers represent the base peak.

#### Organic acids

Organic acids are considered primary metabolites that play critical roles in plant metabolism, including pH modification, metal chelation, and serving as carbon sources for microbes. These diverse functions are vital to the role of these molecules in plant stress biology^34^. Although primarily involved in plant metabolism, many organic acids also confer benefits to human health and skin, including antioxidant activity, anti-aging effects, and support for metabolic and cellular functions^35^. Among these, peaks **2** [*m/z* 133.0139, C_4_H_5_O_5_^–^] and **7** [*m/z* 191.0565, C_7_H_11_O_6_^–^] were putatively annotated as malic acid and quinic acid, respectively. The observed fragment ions at *m/z* 71 for malic acid and *m/z* 129 for quinic acid correspond to [M–H–44–18]^–^, attributed to the sequential loss of CO_2_ (–44) and H_2_O (–18) (Table 1) and (Suppl. Fig. S1 & S2).

#### Phenolic acids

Phenolic acids, a group of nonflavonoid polyphenolic compounds, are commonly subdivided into derivatives of benzoic acid and cinnamic acid^32^. The characteristic losses of CO_2_ (–44), CO (–28), H_2_O (–18), and CH_3_ (–14) are used to identify these compounds^36^ (Table 1).

For instance, peaks **5** [*m/z* 169.0138, C_7_H_5_O_5_^–^] and **9** [*m/z* 167.0345, C_8_H_7_O_4_^–^] exhibited base peaks at *m/z* 125 and 123, respectively, corresponding to [M–H–44]^–^, resulting from the neutral loss of a CO_2_ group (–44). Additionally, fragment ions at *m/z* 107 and 105, respectively, were due to the subsequent loss of H_2_O (–18), and allowed the identification of these peaks as gallic and vanillic acid, respectively (Suppl. Fig. S3 & S4). Peaks **10** [*m/z* 153.0188, C_7_H_5_O_4_^–^] and **21** [*m/z* 179.0351, C_9_H_7_O_4_^–^] displayed the loss of CO_2_ (–44), resulting in base peak fragment ions at *m/z* 109 and 135, respectively, corresponding to [M–H–44]^–^ and were identified as protocatechuic and caffeic acid, respectively (Suppl. Fig. S5 & S6). Peak **18** [*m/z* 457.1331, C_20_H_25_O_12_^–^] exhibited the loss of pentose and hexose units (–132, − 162), yielding a fragment ion at *m/z* 163, followed by CO_2_ loss to produce a base peak fragment ion at *m/z* 119, corresponding to [M–H–162–132–44]^–^. Accordingly, peak **18** was tentatively identified as coumaric acid pentosyl-hexoside (Suppl. Fig. S7).

#### Phenylpropanoids and phenylethanoids

Phenylpropanoids represent a significant constituent of *C. infortunatum* (Table 1). These compounds originate from the shikimate pathway to protect plants from UV radiation, herbivores, and physical damage. They also exhibit several pharmacological properties, such as anti-inflammatory, anti-aging, and antimicrobial effects^7^. The annotated phenylpropanoid (represented mainly by caffeic acid) and phenyl ethanoid moieties were mostly combined with sugar(s) to generate esters and ethers, respectively. Peaks **24** [*m/z* 785.2486, C_35_H_45_O_20_^–^], **29** [*m/z* 653.2071, C_30_H_37_O_16_^–^], **30** [*m/z* 623.1976, C_29_H_35_O_15_^–^], **41** [*m/z* 577.1903, C_28_H_33_O_13_^–^], and **46** [*m/z* 591.2071, C_29_H_35_O_13_^–^] presented base peak fragment ion at *m/z* 161 for caffeoyl moiety, in addition to other fragment ions corresponding to the loss of the attached sugar residues. Eventually, metabolites **24**, **29**, **30**, **41**, and **46** were tentatively assigned as echinacoside, campneoside Ⅰ, verbascoside, salsaside A, and jionoside C, respectively (Suppl. Fig. S8–S12)^7^. Following the same fragmentation pattern, peaks **42** [*m/z* 651.2286, C_31_H_39_O_15_^–^] and **47** [*m/z* 693.2397, C_33_H_41_O_16_^–^] were tentatively identified as martynoside and acetyl martynoside, respectively, both showing a fragment ion at 193 for deprotonated ferulic acid, and a base peak fragment ion at *m/z* 175, corresponding to feruloyl moiety (Suppl. Fig. S13 & S14).

#### Flavonoids

Flavonoids are plant polyphenolic secondary metabolites with a 15-carbon skeleton comprising two phenyl rings and a heterocyclic ring. They support plant growth, pollination, and stress defense. Owing to their bioactive properties, flavonoids have been associated with numerous health benefits, such as antidiabetic, cardioprotective, neuroprotective, antiviral, antibacterial, and anti-aging effects^37 ^. *C. infortunatum* was found to be rich in flavonols (mainly kaempferol and rhamnazin glycosides) and flavones (apigenin and acacetin derivatives) (Table 1). In flavonoid glycosides, the sugar moieties can be readily identified by MS/MS, according to the neutral loss(es) of the attached sugar(s), including hexose (–162), deoxyhexose (or rhamnose, − 146), glucuronic acid (–176), and pentose (–132)^36,38,39^.

For example, rhamnazin hexoside rhamnoside (**27**) [*m/z* 637.1759, C_29_H_33_O_16_^–^] (Suppl. Fig. S15), showed fragment ions 475 and 329, corresponding to [M–H–162]^–^ and [M–H–162–146]^–^ due to the sequential loss of hexose and rhamnose, respectively. Also, peak **48** or acacetin 7-glucuronide [*m/z* 459.0925, C_22_H_19_O_11_^–^] revealed a fragment ion at *m/z* 283, corresponding to [M–H–176]^–^ as a result of loss of glucuronide moiety (–176) and a base peak fragment ion at *m/z* 268 for [M–H–176–15]^–^ (Suppl. Fig. S16). Among the other putatively annotated flavonoids that showed conjugation between sugar and cinnamoyl moieties included kaempferol coumaroyl hexoside (**44**) [*m/z* 593.1310, C_30_H_25_O_13_^–^], apigenin caffeoyl hexoside (**45**) [*m/z* 593.1320, C_30_H_25_O_13_^–^], and apigenin coumaroyl hexoside (**49**) [*m/z* 577.1373, C_30_H_25_O_12_^–^] (Suppl. Fig. S17–S19).

#### Coumarins

Coumarins are a family of benzopyrones, structurally characterized as lactones formed by the fusion of a benzene ring with an α-pyrone ring. They are widely distributed in nature and have potential value in the prevention and treatment of various ailments. Among the coumarins identified was esculetin (**20**) [*m/z* 177.0193, C_9_H_5_O_4_^–^] showing a fragment ion at *m/z* 133, corresponding to [M–H–44]^–^ as a result of neutral CO_2_ loss (Suppl. Fig. S20). Esculetin has been widely used as an antioxidant, anti-inflammatory, antibacterial, and antiviral. It has therapeutic effects in acute and chronic atopic skin inflammation^40^.

#### Oxylipins

Oxylipins are a class of secondary metabolites produced when polyunsaturated fatty acids undergo further conversion or oxidation^41^. These lipid-based molecules are essential signaling molecules, known to mediate several intricate biological processes across several life domains, similar to the action of hormones. Peaks **22** [*m/z* 387.1650, C_18_H_27_O_9_^–^] and **38** [*m/z* 549.1968, C_27_H_33_O_12_^–^] both give rise to a fragment ion at *m/z* 207, indicating the neutral loss of hexose and H_2_O for peak **22**, corresponding to [M–H–162–18]^–^ and the loss of hexose, caffeoyl, and H_2_O for peak **38**, corresponding to [M–H–162*2–18]^–^. These compounds were thus tentatively assigned as 12-hydroxy-jasmonic acid glucoside and 6’-caffeoyl-12-glucosyloxy-jasmonic acid, respectively^30^(Suppl. Fig. S21 & S22).

#### Fatty acids and lipids

Fatty acids are the main structural components of lipids and serve as the essential building blocks of cell membranes^42^. Peak **51** [*m/z* 327.2168, C_18_H_31_O_5_^–^] differed from peak **53** [*m/z* 329.2350, C_18_H_33_O_5_^–^] by 2 *m/z* units, demonstrating the presence of an additional double bond in the former. These peaks were tentatively identified as trihydroxy-octadecadienoic acid and trihydroxy-octadecenoic acid (Table 1). Similarly, peaks **60** [*m/z* 277.2166, C_18_H_29_O_2_^–^] and **61** [*m/z* 279.2322, C_18_H_31_O_2_^–^] were putatively annotated as octadecatrienoic acid (linolenic acid) and octadecadienoic acid (linoleic acid), respectively (Table 1).

Lipids can be classified into several groups, including phospholipids, sulfolipids, sphingolipids, and glycerolipids. In these lipids, the fatty acid acyl group is esterified with either glycerol (in glycerolipids) or phosphoglycerol (in phospholipids), resulting in a range of derivatives. Sulfolipids are a type of lipid where acylglycerol is linked to a sulfated sugar, usually glucose or quinovose^16^. In the negative ESI mode, fragment ions at *m/z* 255 for hexadecanoic acid (16:0), 277 for octadecatrienoic acid (18:3), 281 for octadecenoic acid (18:1), and 293 for hydroxy-octadecatrienoic acid (18:3[OH]) help identify specific fatty acids esterified to the glycerol backbone. Additionally, lipid classes can be more easily distinguished through other diagnostic ions, such as *m/z* 79 (phosphonate), 81 (sulfonate), 153 (phospho-glycerol), and 225 (sulfoquinovose–18, or sulfoquinovosyl)^36^.

For instance, lipids **56** [*m/z* 555.2832, C_25_H_47_O_11_S^–^], **62** [*m/z* 843.5297, C_47_H_79_O_12_S^–^], and **64** [*m/z* 815.4975, C_43_H_75_O_12_S^–^], showed fragment ions at *m/z* 225 (sulfoquinovosyl) and 81 (sulfonate), and were putatively annotated as sulfoquinovosyl monoacyl glycerol (16:0) (Suppl. Fig. S23), sulfoquinovosyl diacyl glycerol (18:2/18:1), and sulfoquinovosyl diacyl glycerol (16:0/18:3) (Table 1).

Peaks **58** [*m/z* 409.2358, C_19_H_38_O_7_P^–^] and **59** [*m/z* 435.2502, C_21_H_40_O_7_P^–^] showed similar fragment ions at *m/z* 79 and *m/z* 153. Additionally, peak **58** exhibited a fragment ion at *m/z* 255, while peak **59** exhibited a fragment ion at *m/z* 281. Consequently, peaks **58** and **59** were tentatively identified as monoacyl phosphoglyceride (16:0) (Suppl. Fig. S24) and monoacyl phosphoglyceride (18:1) (Table 1), respectively^16^.

### Anti-aging activity

#### Anti-collagenase activity

Collagenase is an enzyme that degrades collagen, a key structural protein in the skin that provides firmness and support. Inhibitors of collagenase are valuable in anti-aging applications, as they help preserve collagen levels, promote skin resilience, and maintain a healthy appearance^4^. The inhibitory effects of the fractions against collagenase were assessed using *in vitro *experiments. As illustrated in Table 2, the EA fraction showed the strongest inhibition with an IC_50_ of 12.2 µg/mL, outperforming the reference drug piroxicam (IC_50_ of 33.5 µg/mL). EA fraction was followed by HEX, DM, and BU fractions with IC_50_ values of 20.7, 38.6, and 60.9 µg/mL, respectively. These results demonstrated the superior collagenase inhibitory activity of the EA fraction compared to both the other fractions and the reference drug.

#### Anti-elastase activity

Elastin fibers are essential for maintaining skin structure, and their degradation results in reduced elasticity, contributing to wrinkles and other signs of aging. To combat this, elastase inhibitors are valuable in anti-aging treatments, as they help preserve elastin levels, promoting firmer, more youthful-looking skin^5^. Among the investigated fractions, the EA fraction showed the most potent elastase inhibitory activity, with an IC_50_ of 25.0 µg/mL that was lower than that of the reference drug daidzein (IC_50_ of 30.2 µg/mL). The HEX, DM, and BU fractions also demonstrated certain inhibitory effects, with IC_50_ values of 39.7, 42.2, and 82.4 µg/mL, respectively. These findings highlighted the EA fraction as the most promising candidate for elastase inhibition (Table 2).

**Table 2:** Anti-collagenase and anti-elastase inhibitory activity of the HEX, DM, EA, and BU fractions from the aerial parts extract of *C. infortunatum*, expressed as IC_50_ (µg/mL).

Samples	Anti-collagenase		Anti-elastase	
	IC_50_ (µg/mL)	± SD	IC_50_ (µg/mL)	± SD
HEX	20.7^c^	0.7	39.7^c^	1.5
DM	38.6^d^	1.4	42.2^c^	1.6
EA	12.2^b^	0.4	25.0^b^	1.0
BU	60.9^e^	2.1	82.4^d^	3.2
Piroxicam*	33.5^a^	1.1		
Daidzein*			30.2^a^	1.2

Different superscript letters (a–e) mean statistically significant differences in the same column (*p* < 0.05) by Tukey’s test. Values are expressed as mean ± SD. * Reference drug.

### Multivariate data analysis

A PLS analysis was conducted to investigate the association between the abundance of the identified metabolites (X variables) across the different fractions and the anti-aging activity against aging-related enzymes (Y variables).

#### Correlation with anti-collagenase activity

**Fig. 2:**
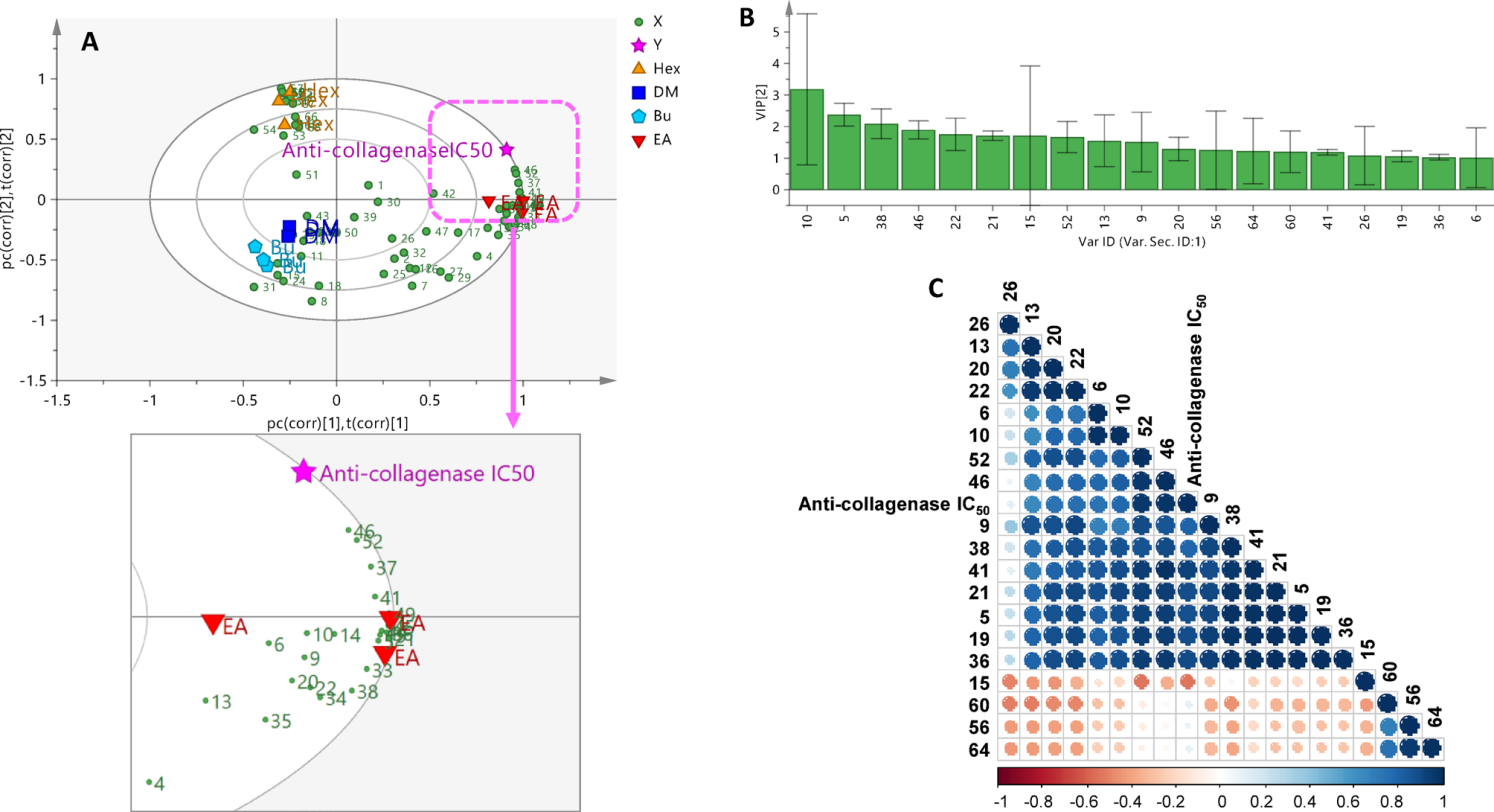
**A**. PLS scores-loadings biplot revealing the correlation of the identified metabolites in *C. infortunatum* fractions with anti-collagenase activity. **B** Metabolites with variable importance to projection (VIP) values ≥ 1. **C** Correlogram visualizing the correlations of the identified metabolites (VIP ≥ 1) with anti-collagenase activity. Correlations with *p*-value > 0.05 are considered non-significant and are represented by a blank white space. The color and size of the circles are proportional to the correlation coefficients. Positive and negative correlations are shown in blue and red colors, respectively. See Table 1 for the metabolite names.

For anti-collagenase activity, the obtained PLS biplot (Fig. 2), which integrates both score and loading plots, revealed a differentiation between the fractions and the variables contributing to their separations. The model exhibited good fitness (R^2^_Y_ cum = 0.99) and high predictive power (Q^2^ cum = 0.97), as supported by validation parameters shown in Suppl. Fig. S25 & S26.

The anti-collagenase activity was projected closest to the EA fraction, suggesting a strong correlation (Fig. 2). This finding was consistent with the *in vitro* test results, where the EA fraction exhibited the highest anti-collagenase activity. To refine the analysis, metabolites with a VIP value ≥ 1 (Fig. 2B) were prioritized, resulting in the selection of 19 metabolites. These were used to construct a correlogram to elucidate their relationship with anti-collagenase activity^19^.

Correlations with a *p*-value > 0.05 were considered non-significant and were represented as blank white spaces in the correlogram (Fig. 2). The color and size of the circles were proportional to the correlation coefficients (r), with negative correlations shown in red (dark red indicating the strongest negative correlation) and positive correlations in shades of blue. The resulting correlogram (Fig. 2C) showed strong correlations for 13 compounds (**5**, **6**, **9**, **10**, **19**, **20**, **21**, **22**, **36**,** 38**,** 41**, **46**, & **52**). Notably, salsaside A (**41**), jionoside C (**46**), and apigenin (**52**) exhibited particularly strong correlations.

#### Correlation with anti-elastase activity

**Fig. 3:**
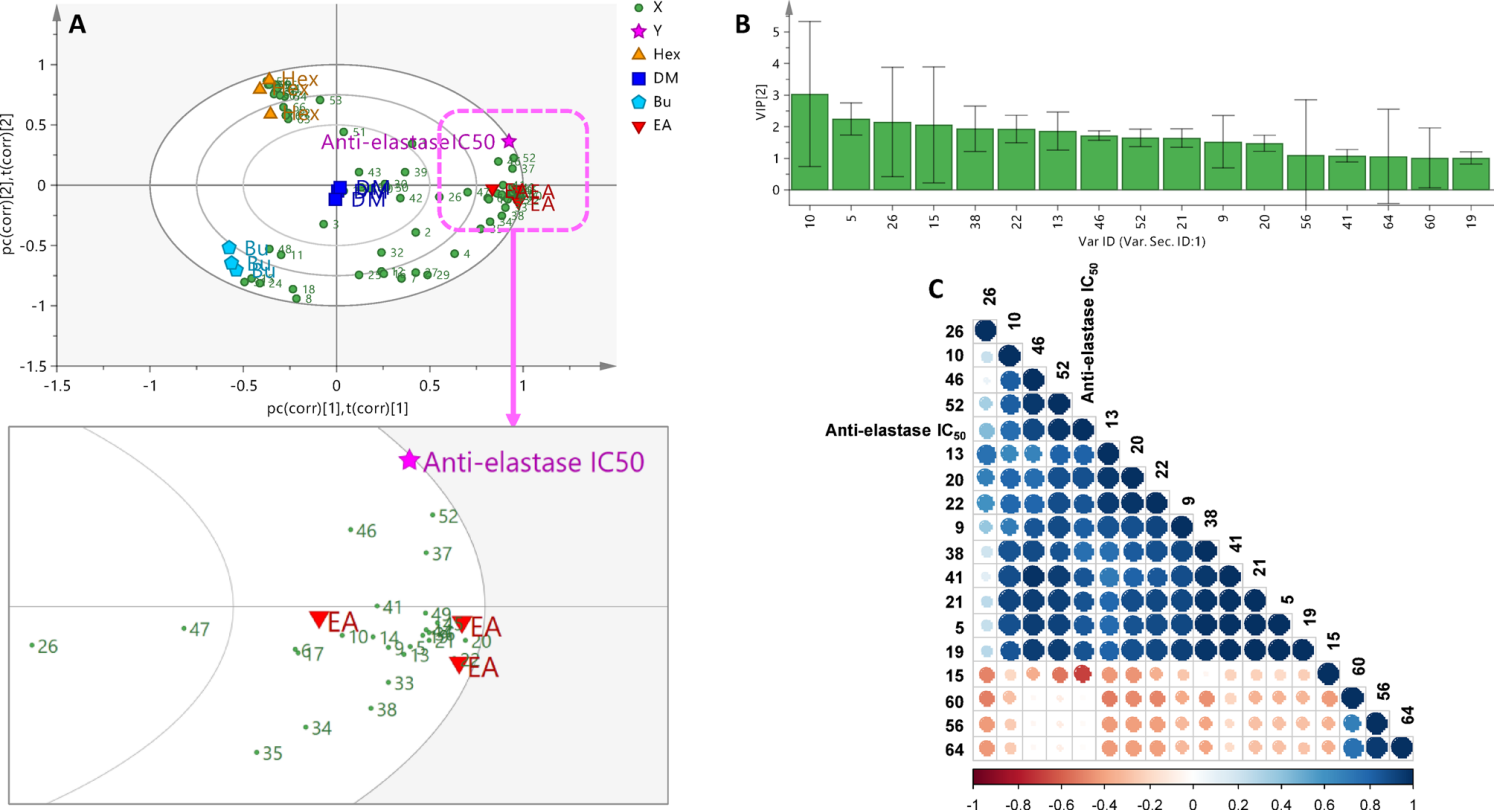
**A** PLS scores-loadings biplot revealing the correlation of the identified metabolites in *C. infortunatum* fractions with anti-elastase activity. **B** Metabolites with variable importance to projection (VIP) values ≥ 1. **C** Correlogram visualizing the correlations of the identified metabolites (VIP ≥ 1) with anti-elastase activity. Correlations with *p*-value > 0.05 are considered non-significant and are represented by a blank white space. The color and size of the circles are proportional to the correlation coefficients. Positive and negative correlations are shown in blue and red colors, respectively. See Table 1 for the metabolite names.

For anti-elastase activity, the PLS biplot (Fig. 3) (R^2^_Y_ cum = 0.99, Q^2^ cum = 0.97, Suppl. Fig. S25 & S26) revealed that the anti-elastase effect was closely correlated to the most active fraction (EA fraction), in agreement with the *in vitro* test results. Selection of metabolites with a VIP value ≥ 1 (Fig. 3B) resulted in a refined list of 17 metabolites for correlogram construction. Among these, 12 metabolites (**5**, **9**, **10**, **13**, **19**, **20**, **21**, **22**, **38**, **41**, **46**, & **52**) showed strong correlations with the anti-elastase activity (Fig. 3C), with jionoside C (**46**) and apigenin (**52**) exhibiting the strongest correlation.

*In vitro* and chemometric studies confirmed that the EA fraction from the aerial parts methanolic extract of *C. infortunatum* exhibited superior inhibitory activity against collagenase and elastase compared to the other fractions and the reference drugs. This enhanced activity was correlated with diverse phytochemical classes, including phenolic acids, flavonoids, phenylpropanoids, phenylethanoids, and other polyphenols, which are well-known for their antioxidant, anti-inflammatory, and anti-aging properties^43^. Additionally, some jasmonate-type oxylipins were also found to be correlated with the investigated activities **(22** & **38**). Jasmonates are known to exhibit antioxidant properties by neutralizing ROS and reducing oxidative stress, which is closely linked to aging, cancer, cardiovascular diseases, and neurodegenerative disorders^41^.

Interestingly, gallic acid (**5**), vanillic acid (**9**), protocatechuic acid (**10**), phenyl-acetic acid (**19**), esculetin (**20**), caffeic acid (**21**), 12-hydroxy-jasmonic acid glucoside (**22**), 6’-caffeoyl-12-glucosyloxy-jasmonic acid (**38**), salsaside A (**41**), jionoside C (**46**), and apigenin (**52**) were strongly correlated with both anti-collagenase and anti-elastase activity. Notably, these metabolites primarily belonged to phenolic acids (**5**, **9**, **10**, and **19**), coumarins (**20**), oxylipins (**22** & **38**), phenylpropanoids (**41** & **46**), and flavonoids (**52**) classes, and were predominantly found in the EA fraction.

Overall, polyphenols are renowned for their broad therapeutic potential, including anti-inflammatory, antitumor, and antimicrobial activities, as well as their ROS scavenging and antioxidant capabilities. Increasing evidence suggests that polyphenols can slow or prevent aging-related deterioration in both the appearance and function of the skin^44^. Among them, gallic acid (**5**) is known for its strong antioxidant and ROS-scavenging abilities. A clinical trial involving a gel enriched with gallic acid and other phenolic compounds derived from *Terminalia chebula* showed significant anti-aging effects in the skin of human volunteers, including improved skin elasticity and reduced roughness^45^. Similarly, caffeic acid (**21**), known for its potent antioxidant properties, promotes collagen production and helps prevent premature skin aging. It also exhibits antimicrobial properties, making it a promising candidate for treating various dermal diseases^46^.

Apigenin (**52**), widely recognized for its multiple biological activities, is used as a dietary supplement and has been shown to reduce skin inflammation by downregulating several inflammatory mediators^47^. Phenylpropanoids and phenylethanoids such as salsaside A and jionoside C (**41** & **46)** are also well-documented for their anti-inflammatory, antimicrobial, anti-skin-aging, and antitumor effects^48^. Coumarins, including esculetin (**20**), have been reported for their antioxidant and anti-inflammatory properties, showing potential for the treatment of numerous diseases associated with inflammation and ROS. In a recent study, esculetin (50–100 mg/kg) significantly decreased the pro-inflammatory cytokine mRNA levels in mouse skin^49^.

### Molecular docking

**Fig. 4:**
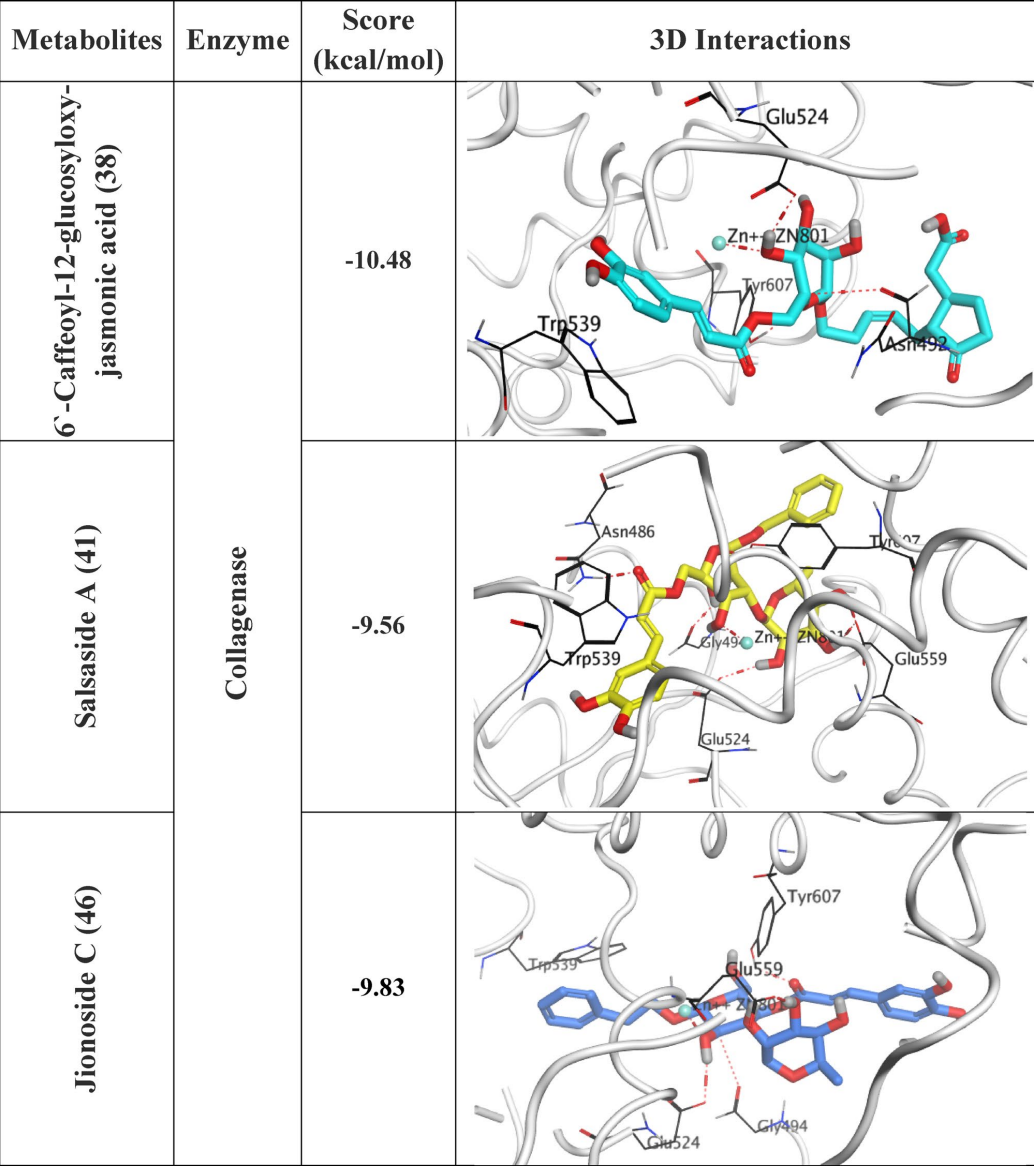
Binding scores and 3D binding interactions of 6′-caffeoyl-12-glucosyloxy-jasmonic acid (**38**), salsaside A (**41**), and jionoside C (**46**) with the collagenase enzyme (PDB ID: 7Z5U).

**Fig. 5:**
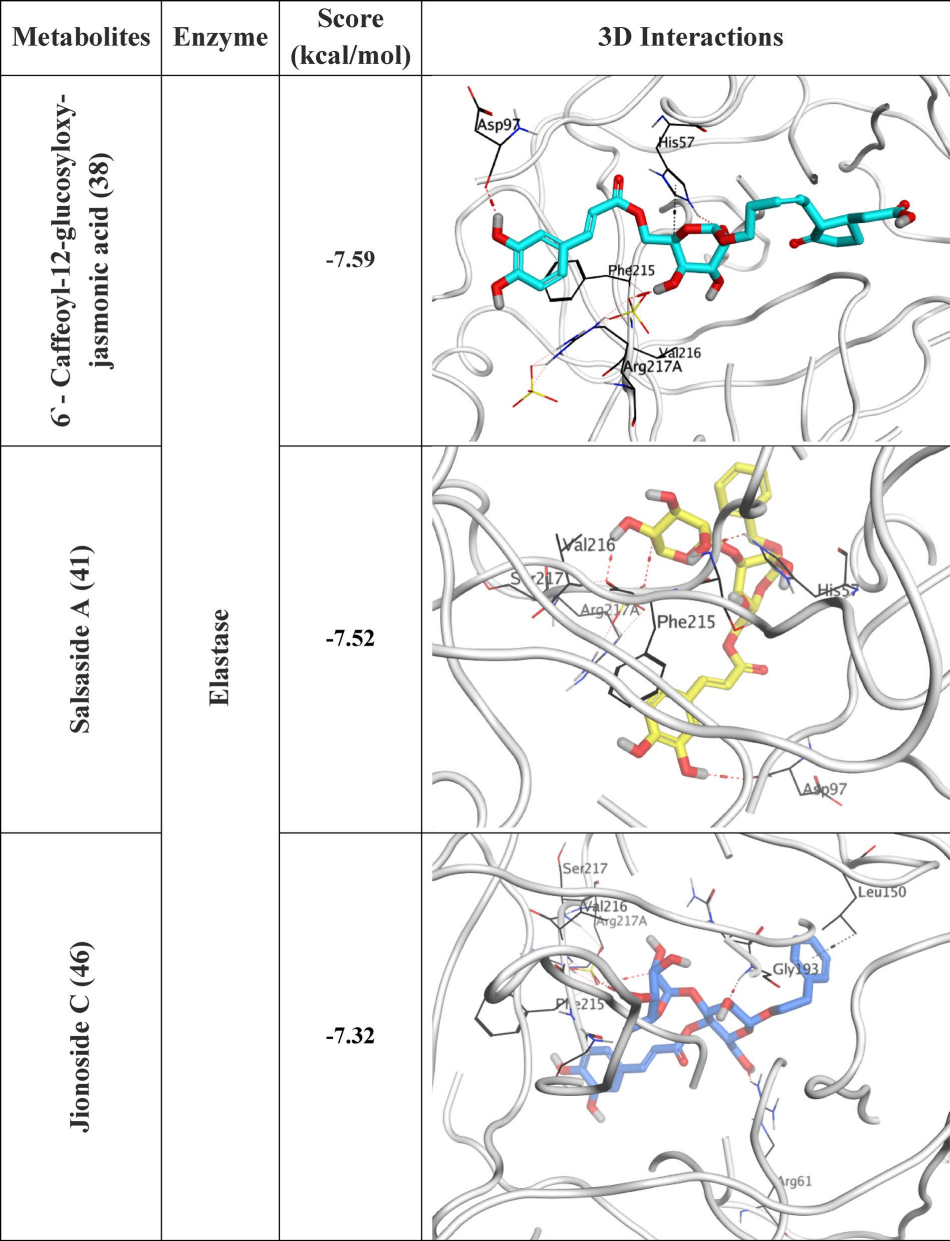
Binding scores and 3D binding interactions of 6′-caffeoyl-12-glucosyloxy-jasmonic acid (**38**), salsaside A (**41**), and jionoside C (**46**) with the elastase enzyme (PDB ID: 4YM9).

The strongly correlated metabolites (**5**, **9**, **10**, **19**, **20**, **21**, **22**, **38**,** 41**, **46**, & **52**) with both elastase and collagenase activities were subjected to molecular docking studies to explore their potential interaction mechanisms and predict their binding modes with the crystallized enzyme structures. Key amino acid residues and Zn^2+^ identified as crucial for binding were Ser195, Gly193, His57, Phe215, Arg217, Ser217, and Val216 in elastase and Tyr607, Gly494, Glu524, His527, Asn486, Glu498, Trp539, and Zn801 in collagenase. 6′-Caffeoyl-12-glucosyloxy-jasmonic acid (**38**), salsaside A (**41**), and jionoside C (**46**) demonstrated the highest binding affinities toward both enzymes. They presented binding scores of − 10.48, − 9.56, and − 9.83 kcal/mol for collagenase, and − 7.59, − 7.52, and − 7.32 kcal/mol for elastase. These values were comparable to, and in some cases exceeded, those of the co-crystallized inhibitors of collagenase (− 10.70 kcal/mol) and elastase (− 5.61 kcal/mol) (Figs. 4 and 5).

For the collagenase target (PDB ID: 7Z5U), 6′-caffeoyl-12-glucosyloxy-jasmonic acid (**38**) displayed three hydrogen bonds with Glu524, Asn492, and Tyr607, in addition to a metal complex with Zn^2+^ 801, and Trp539. Additionally, salsaside A (**41**) interacted *via* six hydrogen bonds involving Glu559 (two bonds), Glu524, Gly494, Tyr607, and Asn486, along with a metal complex with Zn^2+^ 801 and one π-π bond with Trp539. Finally, jionoside C (**46**) formed four hydrogen bonds with Glu559, Glu524, Gly494, and Tyr607, a metal complex with Zn^2+^ 801(Fig. 4), similar to the co-crystallized ligand, and one π-π bond with Trp539.

For the elastase target (PDB ID: 4YM9), 6′-caffeoyl-12-glucosyloxy-jasmonic acid (**38**) engaged through one hydrogen bond and one π-hydrogen bond with His57, one hydrogen bond with Asp97, and one hydrogen bond with the sulphate group linked to Val216, Arg217, and Phe215. Moreover, salsaside A (**41**) showed one hydrogen bond with His57 and two hydrogen bonds with the sulphate group linked to Arg217, Phe215, Ser217, and Val216. Finally, jionoside C (**46**) formed two hydrogen bonds with His57 & Arg61, a π-hydrogen bond with Leu150, and two hydrogen bonds with the sulphate group linked to Ser217, Phe215, Arg217, and Val216 (Fig. 5) (like the co-crystallized ligand).

Accordingly, the molecular docking studies identified 6’-caffeoyl-12-glucosyloxy-jasmonic acid (**38**), salsaside A (**41**), and jionoside C (**46**) as the key metabolites responsible for the observed anti-elastase and anti-collagenase activities. In contrast to other metabolites highlighted by multivariate data analysis as strongly correlated with these bioactivities, these three compounds share a common structural core, the caffeoyl glucoside moiety, which may serve as a novel pharmacophore for the design of future anti-aging therapeutics. For comparison, apigenin showed moderate binding affinity to elastase and collagenase, with scores below − 6.0 and − 8.0 kcal/mol, respectively. This weaker interaction may be attributed to structural differences: while apigenin possesses a flavonoid backbone, the top-scoring compounds share the caffeoyl glucoside moiety, which appears to play a crucial role in mediating binding interactions. Therefore, the modest docking performance of apigenin suggests that its anti-aging effects may not arise from direct inhibition of collagenase or elastase, but rather from alternative mechanisms such as its antioxidant, ROS-scavenging, and anti-inflammatory activities^47^, which are also relevant to skin aging. These findings underscore the importance of integrating multivariate data analysis with molecular docking to capture the multifaceted modes of action exhibited by plant-derived metabolites.

Overall, this study is the first to identify these specific metabolites as potential candidates for anti-aging drug development, derived from EA fractions of methanolic extracts of the aerial parts of *C. infortunatum*. However, the limited number of samples in the chemometric analysis makes the findings preliminary and indicates the need for further studies with larger datasets. Furthermore, molecular docking provides predictive insights that require experimental confirmation and could be extended through molecular simulation to better understand metabolite–target interactions. Additionally, future investigations should include quantitative analysis of major bioactive markers to better establish dose–response relationships, alongside studies exploring their therapeutic potential and validating their safety in clinical applications for aging-related conditions.

## Conclusion

To the best of our knowledge, this is the first study to establish a direct correlation between the secondary metabolites of different solvent-extracted fractions of the aerial parts of *C. infortunatum* and its newly discovered anti-aging properties. LC-MS/MS-based metabolite profiling in negative ionization mode tentatively identified 67 metabolites across these fractions, including organic acids, phenolic acids, phenylpropanoids, phenylethanoids, flavonoids, coumarins, oxylipins, other aromatic compounds, fatty acids and lipids, and miscellaneous compounds. The tested fractions exhibited significant anti-aging activity by inhibiting collagenase and elastase enzymes, with the EA fraction demonstrating the highest activity, surpassing even reference drugs. PLS multivariate data analysis linked this strong activity to the high concentration of polyphenols, particularly phenolic acids, phenylpropanoids, phenylethanoids, flavonoids, and jasmonate-type oxylipins. Molecular docking studies identified 6’-caffeoyl-12-glucosyloxy-jasmonic acid, salsaside A, and jionoside C as key metabolites likely responsible for the observed anti-collagenase and anti-elastase activities, further reinforcing the therapeutic potential of *C. infortunatum* in skin aging management. Given these findings, *C. infortunatum* emerges as a promising natural source of anti-aging compounds, with the identified metabolites serving as potential lead compounds for skincare and pharmaceutical formulations targeting enzymatic degradation of skin proteins. However, further studies are warranted to validate these *in vitro* and molecular docking findings through in vivo assays, providing deeper mechanistic insights and confirming the clinical applicability of *C. infortunatum* extracts in anti-aging therapies.

## Supplementary Information

Below is the link to the electronic supplementary material.

Supplementary Material 1

## Data Availability

The datasets used and/or analyzed during the current study are available from the corresponding author on request.

## Abbreviations

BU: n-Butanol
C.: Clerodendrum
DM: Dichloromethane
EA: Ethyl acetate
HEX: Hexane
LC-QTOF-MS/MS: Liquid chromatography-quadrupole time-of-flight tandem mass spectrometry
MMP: Matrix metalloproteinases
*m/z*: Mass-to-charge ratio
PDB: Protein data bank
PLS: Partial least squares
ROS: Reactive oxygen species
R_t_: Retention time
VIP: Variable importance to projection

## Declarations

### Clinical trial statement

Clinical trial number: Not applicable.
