# Supplementary material for: Integrated metabolite profiling and molecular docking reveal anti-aging potential of Clerodendrum infortunatum L. fractions
Source: Sci Rep. 2026 Apr 22;16:18614. doi: 10.1038/s41598-026-47614-3 (PMC13270036; doi:10.1038/s41598-026-47614-3)
Supplement: Supplementary file 1 — Supplementary Material 1 [file 41598_2026_47614_MOESM1_ESM.docx]

**Supplementary Materials**

**Integrated Metabolite Profiling and Molecular Docking Reveal Anti-Aging Potential of *Clerodendrum infortunatum* L. Fractions**

Fatma Atef^1,2^, Mostafa A. Abdelkawy^1^, Basma M. Eltanany^3^, Laura Pont^4,5^, Ahmed A. Al‐Karmalawy ^6,7^, Fernando Benavente^4*^, Inas Y. Younis^1#^, Asmaa M. Otify^1 *^*^#^*

^1^ *Department of Pharmacognosy, Faculty of Pharmacy, Cairo University, Cairo 11562, Egypt*

^2^ *Boulaq El-dakrour General Hospital, Giza 12617, Egypt*

*^3^Department of Pharmaceutical Analytical Chemistry, Faculty of Pharmacy, Cairo University, Cairo 11562, Egypt*

*^4^Department of Chemical Engineering and Analytical Chemistry, Institute for Research on Nutrition and Food Safety (INSA·UB), University of Barcelona, Barcelona 08028, Spain*

*^5^ Serra Húnter Program, Generalitat de Catalunya, Barcelona 08007, Spain*

*^6^Department of Pharmaceutical Chemistry, College of Pharmacy, The University of Mashreq, Baghdad 10023, Iraq.*

*^7^ Department of Pharmaceutical Chemistry, Faculty of Pharmacy, Horus University-Egypt, New Damietta 34518, Egypt.*

** Corresponding author; Asmaa M. Otify,* [*asmaa.otify@pharma.cu.edu.eg*](mailto:asmaa.otify@pharma.cu.edu.eg)*; Fernando Benavente,* [*fbenavente@ub.edu*](mailto:fbenavente@ub.edu)

*#* *These authors contributed equally to this work.*

**Suppl.Table S1**. Abundance (peak area) of the identified metabolites in the HEX, DM, EA, and BU fractions from the aerial parts extract of *C. infortunatum*, analyzed by LC-QTOF-MS/MS in negative ESI mode. Data is expressed as mean ± SD (n = 3).

| **Peak No.** | **R_t_**  **(min)** | ***m/z*** | **Molecular formula** | **Metabolite name** | **HEX** | **DM** | **BU** | **EA** |
| --- | --- | --- | --- | --- | --- | --- | --- | --- |
| **1** | 1.00 | 341.1080 | C_12_H_21_O_11_^–^ | Disaccharide | 132474.7 ± 10032.8 | 167428.6 ± 5577.3 | 102384 ± 11582.2 | 138490.7 ± 5860 |
| **2** | 1.16 | 133.0139 | C_4_H_5_O_5_^–^ | Malic acid | 107906.4 ± 16452.6 | 174081.9 ± 61041.1 | 146712.8 ± 6021.7 | 169703.3 ± 8463 |
| **3** | 1.32 | 128.0349 | C_5_H_6_NO_3_^–^ | Pyroglutamic acid | 57762.3 ± 16556.8 | 321230.5 ± 26834 | 139389.6 ± 35819.7 | 89205.6 ± 1240.7 |
| **4** | 1.47 | 117.0189 | C_4_H_5_O_4_^–^ | Succinic acid | 87195.6 ± 11025.3 | 178201.2 ± 3465.9 | 593092.9 ± 29230.2 | 911711.7 ± 24898.9 |
| **5** | 1.70 | 169.0138 | C_7_H_5_O_5_^–^ | Gallic acid | 28507 ± 17107.6 | 73709.7 ± 40055.1 | 127454 ± 27998.6 | 3325778.1 ± 244422.7 |
| **6** | 1.72 | 125.0234 | C_6_H_5_O_3_^–^ | Pyrogallol or phloroglucinol | 15548.2 ± 2528 | 23851.6 ± 10116.5 | 39831.3 ± 15378.9 | 723067.8 ± 474535.6 |
| **7** | 1.91 | 191.0565 | C_7_H_11_O_6_^–^ | Quinic acid | 707814.3 ± 19792.5 | 2160781.3 ± 63256.3 | 3643618.7 ± 1761799 | 3666089.6 ± 59996.2 |
| **8** | 2.13 | 279.1076 | C_11_H_19_O_8_^–^ | 2-Hydroxy-2-methyl butyric acid hexoside | 42693.1 ± 10953.4 | 191906.4 ± 7141.7 | 476705.5 ± 11204 | 220806.4 ± 6067.1 |
| **9** | 2.58 | 167.0345 | C_8_H_7_O_4_^–^ | Vanillic acid | 14495.6 ± 5411.7 | 290667.7 ± 36939.2 | 20090 ± 4443.6 | 1557097 ± 581093.5 |
| **10** | 3.40 | 153.0188 | C_7_H_5_O_4_^–^ | Protocatechuic acid | 79092.7 ± 38871.5 | 231521.2 ± 174357.3 | 18870.6 ± 3162 | 6477008.4 ± 3586798.7 |
| **11** | 3.86 | 461.1654 | C_20_H_29_O_12_^–^ | Decaffeoyl-acteoside | 31698.6 ± 12727.3 | 96920.2 ± 68567.2 | 1042662.4 ± 1008028.8 | 202968.4 ± 181086.6 |
| **12** | 5.18 | 285.0607 | C_12_H_13_O_8_^–^ | Catechol hexuronide | 20015.9 ± 6724.8 | 29115.5 ± 9846.9 | 182945 ± 901.6 | 168000.7 ± 55287.7 |
| **13** | 5.87 | 137.0240 | C_7_H_5_O_3_^–^ | Hydroxy-benzoic acid | 90125.9 ± 69286.1 | 1603067.2 ± 486520.2 | 64856.5 ± 29235.1 | 2554472.9 ± 372412.1 |
| **14** | 6.24 | 109.0295 | C_6_H_5_O_2_^–^ | Catechol | 1708.1 ± 632.3 | 13315.4 ± 10392.6 | 10478.1 ± 2350.8 | 568842.8 ± 177125.9 |
| **15** | 6.40 | 487.1446 | C_21_H_27_O_13_^–^ | Cistanoside F | 280885.7 ± 7544.3 | 503710 ± 139488.9 | 3997878.9 ± 386201.3 | 821697.3 ± 19435.9 |
| **16** | 6.81 | 341.0865 | C_15_H_17_O_9_^–^ | Caffeoyl glucose | 47344.3 ± 17041.8 | 64536.1 ± 22320.3 | 710263.9 ± 29278.3 | 646210.2 ± 17146.3 |
| **17** | 7.04 | 151.0400 | C_8_H_7_O_3_^–^ | Methoxy-benzoic acid | 4750.4 ± 840.1 | 89592 ± 20462.2 | 2148.4 ± 1135.5 | 101333.4 ± 27767.2 |
| **18** | 7.28 | 457.1331 | C_20_H_25_O_12_^–^ | Coumaric acid pentosyl- hexoside | 67596.8 ± 8850.1 | 149113.1 ± 14272.4 | 633493.5 ± 32958.4 | 286311.8 ± 26974.6 |
| **19** | 7.42 | 135.0446 | C_8_H_7_O_4_^–^ | Phenyl-acetic acid | 26268.6 ± 3640.3 | 44238.7 ± 13743.7 | 19920 ± 2240 | 658563.9 ± 44018.6 |
| **20** | 7.35 | 177.0193 | C_9_H_5_O_4_^–^ | Esculetin | 69920.2 ± 7039.8 | 742338.7 ± 14350.5 | 7891.2 ± 2467 | 1434329.9 ± 39474.2 |
| **21** | 7.46 | 179.0351 | C_9_H_7_O_4_^–^ | Caffeic acid | 73040.9 ± 11696.7 | 133248.6 ± 52221.1 | 76356.6 ± 3953.6 | 1757028.4 ± 128121.3 |
| **22** | 7.52 | 387.1650 | C_18_H_27_O_9_^–^ | 12-Hydroxy-jasmonic acid glucoside | 821645.1 ± 18461 | 1834200.3 ± 127801.3 | 854774.8 ± 419313.2 | 3291636.7 ± 112319.8 |
| **23** | 7.58 | 121.0292 | C_7_H_5_O_2_^–^ | Benzoic acid | 84582.3 ± 23127.8 | 2225769.3 ± 117964.4 | 11669.9 ± 1599.4 | 118503.6 ± 30107.2 |
| **24** | 7.87 | 785.2486 | C_35_H_45_O_20_^–^ | Echinacoside | 11260.4 ± 1010.1 | 30102.3 ± 6287 | 177933 ± 12688.7 | 43328.2 ± 12772.7 |
| **25** | 7.93 | 639.1917 | C_29_H_35_O_16_^–^ | β-Hydroxy-verbascoside | 252248.5 ± 23560.7 | 528294.5 ± 35463 | 2306127.7 ± 942710.6 | 1761849.9 ± 38881.9 |
| **26** | 8.15 | 225.1127 | C_12_H_17_O_4_^–^ | 12-Hydroxy-jasmonic acid | 448003.8 ± 7563.8 | 12210891.5 ± 160940.4 | 141128.7 ± 6935.6 | 7044380.4 ± 374747.7 |
| **27** | 8.31 | 637.1759 | C_29_H_33_O_16_^–^ | Rhamnazin hexoside rhamnoside | 32758.7 ± 1644.5 | 101916.3 ± 6392 | 438981.2 ± 22445.1 | 486962 ± 8248.6 |
| **28** | 8.43 | 167.0343 | C_8_H_7_O_4_^–^ | Dihydroxy-methyl benzoate | 81377.5 ± 10068.3 | 1052832.1 ± 12604.9 | 1486.8 ± 330.8 | 166505.1 ± 10432.1 |
| **29** | 8.49 | 653.2071 | C_30_H_37_O_16_^–^ | Campneoside Ⅰ | 385954.6 ± 11097.2 | 727214.4 ± 34936.3 | 1488509.8 ± 30138.2 | 1698345.3 ± 73816.4 |
| **30** | 8.64 | 623.1976 | C_29_H_35_O_15_^–^ | Verbascoside | 4894273.7 ± 2589419.4 | 5289570.9 ± 2608293.3 | 4626227.6 ± 462532.7 | 5904950.6 ± 2243488.2 |
| **31** | 8.82 | 755.2377 | C_34_H_43_O_19_^–^ | Forsythoside B | 15414 ± 4974.1 | 30828.9 ± 4991.1 | 59769 ± 10510.6 | 19876 ± 1980.6 |
| **32** | 8.91 | 623.1978 | C_29_H_35_O_15_^–^ | Iso-verbascoside | 3198097.7 ± 199756.8 | 3405072.8 ± 1746907.3 | 7337735.6 ± 4024109.2 | 7302932.7 ± 3041000.1 |
| **33** | 9.05 | 607.2031 | C_29_H_35_O_14_^–^ | Lipedoside A-Ⅰ | 95530.2 ± 11904.5 | 121257.2 ± 6999.1 | 175258 ± 5808.4 | 683825.8 ± 4821 |
| **34** | 9.17 | 637.2126 | C_30_H_37_O_15_^–^ | Leucosceptoside A | 164892 ± 3520.3 | 166240.3 ± 7489.5 | 351417.2 ± 8280.6 | 826110.1 ± 61101.4 |
| **35** | 9.17 | 785.2278 | C_38_H_41_O_18_^–^ | Caffeoyl-verbascoside | 24645.8 ± 5602.6 | 42630.8 ± 24949.9 | 143069.7 ± 58040.4 | 348311.4 ± 19233.5 |
| **36** | 9.30 | 503.1187 | C_24_H_23_O_12_^–^ | Dicaffeoyl hexoside | 33309.1 ± 4457.1 | 47833.2 ± 5581.2 | 19469 ± 3556.8 | 618875.1 ± 14782.1 |
| **37** | 9.29 | 431.0971 | C_21_H_19_O_10_^–^ | Apigenin hexoside | 109577.3 ± 5261.2 | 96544.7 ± 3295.6 | 69079.1 ± 3928.9 | 238895.3 ± 13943.4 |
| **38** | 9.46 | 549.1968 | C_27_H_33_O_12_^–^ | 6ʹ-Caffeoyl-12-glucosyloxy-jasmonic acid | 256330.4 ± 3051.5 | 505872.8 ± 23682.5 | 930389.3 ± 76303.4 | 3525013.8 ± 50065.7 |
| **39** | 9.56 | 187.0970 | C_9_H_15_O_4_^–^ | Nonanedioic acid (Azelaic acid) | 233448.7 ± 4912.9 | 1018805.9 ± 15942.2 | 9495.6 ± 1365.5 | 435056.4 ± 15064.9 |
| **40** | 9.73 | 243.1240 | C_12_H_19_O_5_^–^ | Trihydroxy-dodecadienoic acid | 23538.2 ± 10788.8 | 318706.6 ± 9020.8 | 3571.1 ± 844.6 | 69057.6 ± 21664.1 |
| **41** | 9.76 | 577.1903 | C_28_H_33_O_13_^–^ | Salsaside A | 190553.6 ± 8778.7 | 67361.1 ± 3381.7 | 128046.5 ± 3912.6 | 850349.7 ± 19934.8 |
| **42** | 9.85 | 651.2286 | C_31_H_39_O_15_^–^ | Martynoside | 724046.5 ± 40605.6 | 208968.2 ± 163103.8 | 914815.4 ± 46980.4 | 1266109.5 ± 673967.4 |
| **43** | 9.92 | 193.0501 | C_10_H_9_O_4_^–^ | Ferulic acid | 263913 ± 4379.2 | 1210740.5 ± 32614.1 | 10542 ± 2763.4 | 244689.8 ± 4230 |
| **44** | 10.00 | 593.1310 | C_30_H_25_O_13_^–^ | Kaempferol coumaroyl-hexoside | 10675.8 ± 2412.5 | 16001.5 ± 3592.2 | 4754.1 ± 1130.4 | 217926.9 ± 13649.8 |
| **45** | 10.20 | 593.1320 | C_30_H_25_O_13_^–^ | Apigenin caffeoyl-hexoside | 25761.2 ± 6365.6 | 39545.5 ± 4680.9 | 3759.1 ± 685.3 | 451801.7 ± 20319.4 |
| **46** | 10.24 | 591.2071 | C_29_H_35_O_13_^–^ | Jionoside C | 697421.8 ± 14653.2 | 177397.1 ± 9204.6 | 184583.8 ± 17285.6 | 1902796.2 ± 49712.2 |
| **47** | 10.54 | 693.2397 | C_33_H_41_O_16_^–^ | Acetyl martynoside | 52816.5 ± 7043.7 | 496905.2 ± 17221.3 | 6443.9 ± 4106.3 | 387584.4 ± 15095.3 |
| **48** | 10.63 | 459.0925 | C_22_H_19_O_11_^–^ | Acacetin 7- glucuronide | 196182.5 ± 6824 | 84697.5 ± 3946.3 | 502299.1 ± 209364 | 224033.8 ± 10276.6 |
| **49** | 10.78 | 577.1373 | C_30_H_25_O_12_^–^ | Apigenin coumaroyl-hexoside | 17335.5 ± 1692.7 | 11192.2 ± 3484.5 | 1367.8 ± 461.7 | 210916.3 ± 10431.1 |
| **50** | 10.89 | 299.0544 | C_16_H_11_O_6_^–^ | 4-Methyl scutellerein | 33614 ± 16005.5 | 209046.5 ± 5655 | 15941.6 ± 1504 | 72126.1 ± 5592.6 |
| **51** | 11.20 | 327.2168 | C_18_H_31_O_5_^–^ | Trihyroxy-octadecadienoic acid | 1481019.9 ± 34202.4 | 2738692.5 ± 121039.5 | 19212.1 ± 2002.2 | 687986.7 ± 193760.6 |
| **52** | 11.45 | 269.0458 | C_15_H_9_O_5_^–^ | Apigenin | 520381 ± 9735.7 | 409138 ± 2672.7 | 12380.5 ± 6024.4 | 1526703.1 ± 33704.3 |
| **53** | 11.62 | 329.2350 | C_18_H_33_O_5_^–^ | Trihydroxy-octadecenoic acid | 1107288.4 ± 13481 | 1239413.6 ± 71553.4 | 14384 ± 1629.2 | 332878.2 ± 1388.3 |
| **54** | 13.32 | 283.0605 | C_16_H_11_O_5_^–^ | Acacetin | 1549419.3 ± 33255.9 | 1494743.5 ± 33979.7 | 43407.2 ± 4687.6 | 151255 ± 997.7 |
| **55** | 13.81 | 675.3588 | C_33_H_55_O_14_^–^ | Dihexosyl monoacyl glycerol (18:3) | 277010.4 ± 66901.1 | 5487.3 ± 1975.1 | 7675.3 ± 618.7 | 19253.5 ± 7764.1 |
| **56** | 13.91 | 555.2832 | C_25_H_47_O_11_S^–^ | Sulfoquinovosyl monoacyl glycerol (16:0) | 4176698.2 ± 3009774.4 | 121908.6 ± 7536.8 | 119559.3 ± 3358.3 | 47541.2 ± 25916.6 |
| **57** | 15.93 | 295.2277 | C_18_H_31_O_3_^–^ | Hydroxy-octadecadienoic acid | 1741907.4 ± 156595.5 | 38904 ± 7012.1 | 4058.4 ± 1989 | 9163.1 ± 1767.3 |
| **58** | 17.45 | 409.2385 | C_19_H_38_O_7_P^–^ | Monoacyl phosphoglyceride (16:0) | 461956.5 ± 111763 | 7839.2 ± 2288.1 | 2331.4 ± 848.6 | 5386.7 ± 3492.3 |
| **59** | 17.87 | 435.2502 | C_21_H_40_O_7_P^–^ | Monoacyl phosphoglyceride (18:1) | 105671.8 ± 28108.6 | 2440.6 ± 919.6 | 676.8 ± 368.5 | 1386 ± 481.6 |
| **60** | 18.58 | 277.2166 | C_18_H_29_O_2_^–^ | Octadecatrieoic acid | 3146131.5 ± 720029.8 | 27379.4 ± 6019.3 | 1262.3 ± 765.3 | 6711.4 ± 1297.6 |
| **61** | 19.63 | 279.2322 | C_18_H_31_O_2_^–^ | Linoleic acid | 580740.8 ± 277090.7 | 5293 ± 3739.3 | 1752.6 ± 765.7 | 1810.3 ± 696.4 |
| **62** | 19.69 | 843.5297 | C_45_H_79_O_12_S^–^ | Sulfoquinovosyl diacyl glycerol (18:1/18:2) | 84512 ± 105498.4 | 982.2 ± 897.7 | 642.1 ± 20.1 | 769.2 ± 366.6 |
| **63** | 20.85 | 255.2323 | C_16_H_31_O_2_^–^ | Hexadecanoic acid | 344830 ± 331073.9 | 12024 ± 8702.2 | 21883.5 ± 8255.2 | 21965.5 ± 2663.3 |
| **64** | 21.02 | 815.4975 | C_43_H_75_O_12_S^–^ | Sulfoquinovosyl diacyl glycerol (16:0/18:3) | 3830151.7 ± 2592140.1 | 2843.3 ± 2049.7 | 864.3 ± 351.9 | 848.4 ± 414.9 |
| **65** | 21.04 | 281.2478 | C_18_H_33_O_2_^–^ | Octadecenoic acid (Oleic acid) | 263512 ± 248447.4 | 2030.3 ± 1159.3 | 2413.9 ± 410 | 2200.6 ± 694.2 |
| **66** | 21.12 | 841.5126 | C_45_H_77_O_12_S^–^ | Sulfoquinovosyl diacyl glycerol (18:1/18:3) | 279375.7 ± 295518.6 | 675.4 ± 427.3 | 0 ± 0 | 430.2 ± 51.4 |
| **67** | 22.43 | 819.5287 | C_43_H_79_O_12_S^–^ | Sulfoquinovosyl diacyl glycerol (16:0/18:1) | 86575 ± 73673.2 | 2209.3 ± 542.3 | 1076.6 ± 645.4 | 1361.6 ± 655 |

**Suppl. Fig. S1.** MS/MS spectrum of **malic acid** (**2**, Table **1**)

**Suppl. Fig. S2.** MS/MS spectrum of **quinic acid** (**7**, Table **1**)

**Suppl. Fig. S3.** MS/MS spectrum of **gallic acid** (**5**, Table **1**)

**Suppl. Fig. S4.** MS/MS spectrum of **vanillic acid** (**9**, Table **1**)

**Suppl. Fig. S5.** MS/MS spectrum of **protocatechuic acid** (**10**, Table **1**)

**Suppl. Fig. S6.** MS/MS spectrum of **caffeic acid** (**21**, Table **1**)

**Suppl. Fig. S7.** MS/MS spectrum of **coumaric acid pentosyl-hexoside** (**18**, Table **1**)

**Suppl. Fig. S8.** MS/MS spectrum of **echinacoside** (**24,** Table **1**)

**Suppl. Fig. S9.**MS/MS spectrum of **campneoside I** (**29,** Table **1**)

**Suppl. Fig. S10.** MS/MS spectrum of **verbascoside** (**30,** Table **1**)

**Suppl. Fig. S11.** MS/MS spectrum of **salsaside A** (**41**, Table **1**)

**Suppl. Fig. S12.** MS/MS spectrum of **jionoside C** (**46,** Table **1**)

**Suppl. Fig. S13.** MS/MS spectrum of **martynoside** (**42**, Table **1**)

**Suppl. Fig. S14.** MS/MS spectrum of **acetyl martynoside** (**47**, Table **1**)

**Suppl. Fig. S15.** MS/MS spectrum of **rhamnazin hexoside-rhamnoside** (**27**, Table **1**)

**Suppl. Fig. S16.** MS/MS spectrum of **acacetin 7-glucuronide** (**48**, Table **1**)

**Suppl. Fig. S17.** MS/MS spectrum of **kaempferol coumaroyl-hexoside** (**44**, Table **1**)

**Suppl. Fig. S18.** MS/MS spectrum of **apigenin caffeoyl-hexoside** (**45**, Table **1**)

**Suppl. Fig. S19.** MS/MS spectrum of **apigenin coumaroyl-hexoside** (**49**, Table **1**)

**Suppl. Fig. S20.** MS/MS spectrum of **esculetin** (**20**, Table **1**)

**Suppl. Fig. S21.** MS/MS spectrum of **12-hydroxy-jasmonic acid glucoside** (**22**, Table **1**)

**Suppl. Fig. S22.** MS/MS spectrum of **6ʹ-caffeoyl-12-glucosyloxy-jasmonic acid** (**38**, Table **1**)

**Suppl. Fig. S23.** MS/MS spectrum of **sulfoquinovosyl monoacyl glycerol (16:0)** (**56**, Table **1**)

**Suppl. Fig. S24.** MS/MS spectrum of **monoacyl phosphoglyceride (16:0)** (**58**, Table **1**)

**Suppl. Fig. S25.** PLS optimization and validation parameters.The diagnostic metrics R^2^_Y_ cum and Q^2^ cum as a function of the number of principal components for anti-collagenase (**A**) and anti-elastase (**B**) activities of *C. infortunatum* fractions. Cross-validation (CV-ANOVA) results demonstrating to assess the model significance for anti-collagenase (**C**) and anti-elastase (**D**) activities.

**Suppl. Fig. S26.** Relationship between observed and predicted values for (**A**) anti-collagenase and (**B**) anti-elastase activities of *C. infortunatum* fractions. Permutation tests (n = 200) for (**C**) anti-collagenase and (**D**) anti-elastase activities of *C. infortunatum* fractions showing negative Q^2^ intercept values, confirming the model validity.
